# Intraoperative neuromonitoring for function-guided resection differs for supratentorial motor eloquent gliomas and metastases

**DOI:** 10.1186/s12883-015-0476-0

**Published:** 2015-10-20

**Authors:** Thomas Obermueller, Michael Schaeffner, Ehab Shiban, Doris Droese, Chiara Negwer, Bernhard Meyer, Florian Ringel, Sandro M. Krieg

**Affiliations:** Department of Neurosurgery, Klinikum rechts der Isar, Technische Universität München, Ismaninger Str. 22, 81675 Munich, Germany; Department of Anesthesiology, Klinikum rechts der Isar, Technische Universität München, Ismaninger Str. 22, 81675 Munich, Germany

**Keywords:** Glioma, MEP, Metastases, Neuromonitoring, Paresis

## Abstract

**Background:**

Recent data show differences in intraoperative neuromonitoring (IOM) in relation to the operated brain lesion. Due to the recently shown infiltrative nature of cerebral metastases, this work investigates the differences of IOM for cerebral metastases and glioma resection concerning sensitivity, specificity, and predictive values when aiming on preservation of motor function.

**Methods:**

Between 2006 and 2011 we resected 171 eloquently located tumors (56 metastases, 115 gliomas) associated with the rolandic cortex or the pyramidal tract using IOM via direct cortical motor evoked potentials (MEPs). Postoperatively, MEP data were re-analyzed with respect to surgery-related paresis, residual tumor, and postoperative MRI with two different thresholds for MEP decline (50 and 80 % below baseline).

**Results:**

MEP monitoring was successful in 158 cases (92.4 %). MEPs were stable in 54.7 % of all metastases cases and in 65.2 % of all glioma cases (*p* < 0.0001). After metastases resection, 21.4 % of patients improved and 21.9 % deteriorated in motor function. Glioma patients improved in only 5.4 % and worsened in 31.3 % of cases (*p* < 0.05). Resection was stopped due to MEP decline in 8.0 % (metastases) and 34.8 % of cases (gliomas) (*p* < 0.0002).

**Conclusion:**

There is significant difference between glioma and metastases resection. Post-hoc, metastases show more stable MEPs but a surprisingly high rate of surgery-related paresis and therefore a higher rate of false negative IOM.

## Background

For resection of gliomas within or adjacent to the motor system intraoperative neuromonitoring (IOM) is now widely used by many neurosurgeons [1–6]. Although surgery is currently limited to a subgroup of patients harboring brain metastases (BM), especially those symptomatic with a focal deficit are still considered for surgical resection in order to achieve early recovery from neurological deficits [7, 8]. Therefore, particularly metastases within or adjacent to the rolandic cortex or corticospinal tract (CST) are potentially treated by surgical resection. As current reports have shown the infiltrative nature of cerebral metastases, IOM of the motor systems might be helpful to reduce surgery-related motor deficits also in BM patiens and its use therefore increased recently [3, 9–12].

Studies on IOM by motor evoked potentials (MEP) for the resection of gliomas within the rolandic region or CST used various warning criteria with various predictive values even within a considerably homogeneous entity like gliomas [13–17].

The present study, therefore provides data on IOM of a large cohort of motor eloquently located tumors, but also provides a profound analysis of IOM data, which elucidates potential differences of IOM for surgery of supratentorial metastases compared to gliomas as also published earlier by our group. We especially focused on sensitivity, specificity, and predictive values of different warning criteria for each tumor type. Awareness has to be raised among neurosurgeons for these differences when performing surgery in motor eloquent cortex or fiber tracts for these tumor entities.

## Methods

### Patients

Between 2006 and 2011 171 consecutive patients with motor eloquently located supratentorial tumors underwent craniotomy in our department. All of our patients were operated by one of 5 experienced neurosurgeons. There were 56 brain metastases and 115 gliomas in or adjacent to the rolandic cortex or the CST. All cases were performed by monopolar direct cortical stimulation for monitoring of MEPs. Topographic association between tumor and CST or rolandic cortex and therefore indication for IOM was preoperatively assessed by magnetic resonance imaging (MRI). Moreover, prior to surgery, each case was discussed on a case-by-case basis by an interdisciplinary tumor board. Eligibility for surgery was consented by all participating disciplines (neurosurgery, neurooncology, radiation oncology, medical oncology) according to the present guidelines and recent scientific evidence [19–23]. Concerning brain metastases, surgical resection was frequently recommended for patients presenting with disabling motor weakness or lesions resistant to chemo- or radiotherapy. The enrolled patient cohort is outlined in Table 1.

**Table 1:** Enrolled patients

		Metastases	Gliomas
Sex	male	32 (61.0 %)	62 (59.1 %)
	female	21 (39.0 %)	43 (40.9 %)
Age (years)	Mean	61.0.	53.3
	Median	63.0	53.7
	Min	24.2	16.0
	Max	89.4	84.3
Type of primary cancer/WHO-grade	lung cancer	17 (30.0 %)	
	breast cancer	12 (21.0 %)	
	melanoma	5 (9.0 %),	
	colon	4 (7.0 %)	
	renal cancer	4 (7.0 %)	
	others	14 (26.0 %)	
	WHO I	-	3 (2.7 %)
	WHOII	-	17 (15.2 %)
	WHO III	-	22 (19.6 %)
	WHO IV	-	70 (62.5 %)
Number	1	32 (57.0 %)	103 (100 %)
	2	10 (18.0 %)	
	3	6 (11.0 %)	
	>3	8 (14.0 %)	
location of tumor	frontodorsal/SMA	27.0 %	32.0 %
	precentral gyrus	37.0 %	16.0 %
	postcentral gyrus	14.0 %	18.0 %
	CST	22.0 %	34.0 %
preoperative status	paresis	32 (57.0 %)	35 (31.3 %)
	seizures	17 (30.0 %)	24 (21.4 %)
	dizziness	5 (9.0 %)	-
	incidental	2 (4 %)	-
	hemihypesthesia	-	19 (17.0 %)
MEP changes	Intraoperative stable posthoc analysis >50 % amplitude loss	29 (54.7 %)	73 (65.2 %)
	none (stable)	32 (60.0 %)	85 (80.1 %)
	reversible	7 (13.0 %)	9 (8.6 %)
	irreversible	14 (27.0 %)	11 (10.3 %)
	>80 % amplitude loss		
	none (stable)	30 (56.6 %)	24 (22.9 %)
	reversible	20 (37.7 %)	70 (66.7)
	irreversible	3 (5.7 %)	11 (10.3 %)
surgery-related deficits	temporary	5 (9.4 %)	19 (18.1 %)
	permanent	7 (12.5 %)	14 (12.5 %)
Follow-up (months)	Mean ± SD	2.9 ± 4.5	9.7 ± 10.5
	Median	0.7 months	6.1
	Min	0.1	0.5
	Max	20.1	40.6

Overview of all enrolled patients including primary tumor, MEP changes intraoperatively, and surgery-related deficits

### Ethical standard

The study is in accordance with ethical standards of the Declaration of Helsinki and was approved by the local institutional review board (IRB) of the Technical University Munich. The need for consent was waived by the IRB (registration number: 2826/10).

### Standardized patient evaluation

**Fig. 1:**
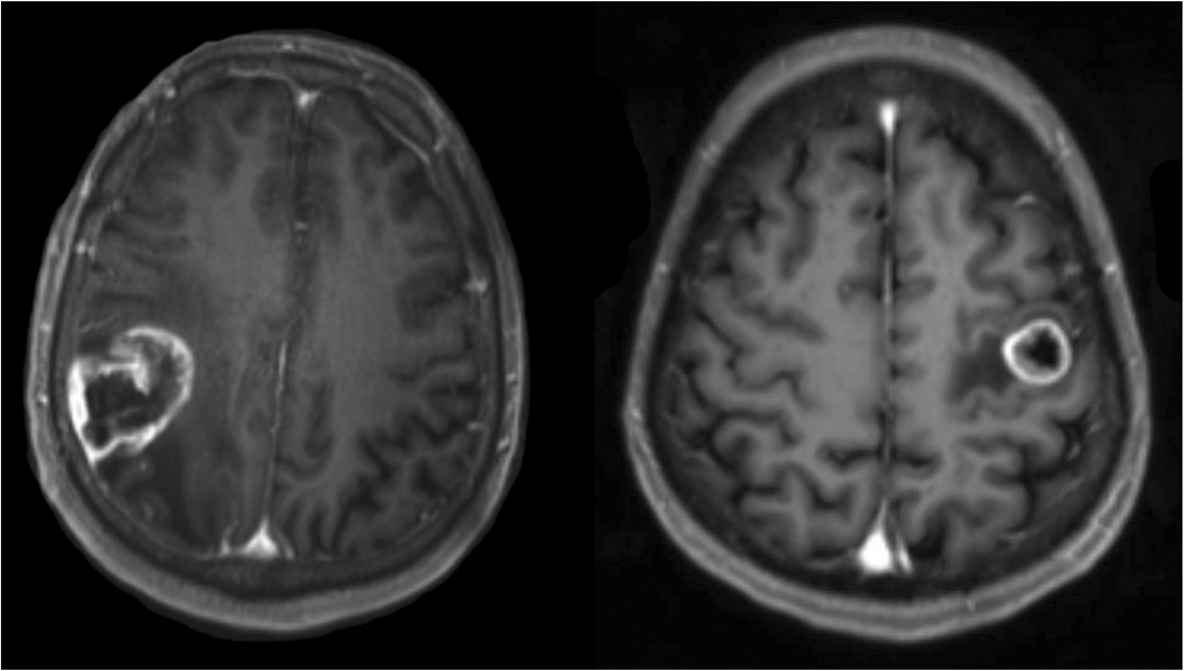
Illustrative MRIs. Two T1 weighted contrast-enhanced MR images showing motor eloquently located glioma within the central sulcus (left) and metastasis in the precentral gyrus (right) as enrolled in this analysis

Preoperatively, 170 out of 171 patients underwent MRI for tumor diagnosis, localization, and acquisition of a navigational dataset for intraoperative neuronavigation (BrainLAB Vector Vision 2^®^, BrainLAB Vector Vision Sky, and BrainLAB Curve^®^, Feldkirchen, Germany) (Figure 1). All patients were evaluated for muscle strength, coordination, sensory function, and cranial nerve function according to a standardized protocol as described earlier [18]. Every patient received steroids preoperatively as part of our clinical standards. Muscle strength was graded according to the British Medical Research Council Scale (BMRC).

Directly after anesthesia all patients were examined routinely and further assessments were performed from the first postoperative day until discharge. During follow-up, first at 6–8 weeks after surgery, and on a regular basis every 3–12 months this standardized protocol was conducted again. On basis of these data, temporary and permanent new surgery-related motor deficits were defined. A new permanent deficit was defined as a new or aggravated paresis due to resection that did not resolve to the preoperative status during follow-up (<6-8 weeks after surgery). A temporary deficit was defined as a new or aggravated postoperative paresis, which disappeared at least during the regular 8-week follow-up. Concerning postoperative imaging, all patients who presented with a new paresis immediately after surgery underwent a postoperative CT scan to exclude to secondary hemorrhage or ischemia. These events were regarded as secondary events, which could not be detected by IOM and therefore were excluded from subset analysis. Additionally, an MRI scan was performed within 48 h after surgery to assess the extent of tumor resection, potential diffusion impairment, increasing edema, or hemorrhage. These data were also reviewed during this study in order to find causes for potentially new postoperative deficits. During follow-up, MRI scans were also performed on a regular basis every 3–12 months depending on tumor entity and current adjuvant therapy. Follow-up MRI scans were reviewed for recurrent metastases and gliomas since the neurological status for this study was only considered during progression-free follow-up.

### Intraoperative monitoring and resection procedure

All patients underwent surgical resection under total intravenous anesthesia (TIVA) by continuously administration of propofol and remifentanyl and volatile anesthetics were strictly avoided due to their interference with evoked potentials [16, 24, 25]. Neuromuscular blocking by rocuronium was used for intubation only and was avoided during surgery for the same reason. Continuous IOM by MEP monitoring was performed by a strip electrode with four to eight contacts (ADTech^®^ strip electrode, AD Technic, City, WI, USA or Inomed Medizintechnik, Emmendingen, Germany), which was positioned onto the hand knob of the precentral gyrus. Subcortical stimulation was only used if needed in this particular surgical step. Somatosensory evoked potential phase reversal or preoperative mapping by navigated transcranial magnetic stimulation confirmed the location of the pre- and postcentral gyrus and the central sulcus [26–28]. For detection of compound muscle action potentials (CMAPs), 27-gauge disposable subdermal needle electrodes (AD-Tech needle electrode, AD-Tech, or Inomed needle electrode, Inomed Medizintechnik) were placed in a bipolar way with a distance of approximately 10 mm over relevant muscles of the contralateral side of the tumor in a standardized way: the thenar (abductor pollicis brevis), hypothenar (adductor digiti minimi), flexor carpi radialis, and brachial biceps for the upper extremity and the anterior tibial muscle for the lower extremity. Processing of the obtained data was achieved by the Axon EpocheXPe neuromonitoring system (Axon Systems, Hauppauge, New York) or the Inomed ISIS IOM system (Inomed Medizintechnik).

For stimulation, square-wave pulses with a duration of 200 to 300 microseconds, a frequency of 350 Hz, and a train of 5 pulses were applied. Stimulation intensity began with 6 mA and was increased continuously in steps of 1 mA until we were able to record a CMAP or until an upper limit of 30 mA was applied without eliciting a CMAP.

Every 2 to 60 s, sequential MEP monitoring was then performed by anodal direct cortical or subcortical stimulation of the rolandic cortex or CST until end of resection and before dural closure extensively described in earlier reports [2, 17, 29]. MEPs were continuously recorded and analyzed in real-time and monitored for amplitude and latency at least every 60 s by a technologist trained as a neurophysiologist or a neurosurgeon with certification in IOM. Depending on the status of the resection, intervals were shortened to 2 s to assure maximum safety during critical steps of resection. The MEP amplitude was evaluated by measuring peak-to-peak differences, whereas latency was defined as the span between the sequence start of the stimulation and the first assessable amplitude as also reported by other groups [17, 23, 29, 30]. During surgery, an amplitude decrease of 50 % or more of the baseline was considered a considerable decline and was immediately reported to the surgeon if not caused by technical issues. However, final consideration of the IOM data was left to the operating surgeon. He or she then reversed the supposed underlying surgical step, removed spatulas, or irrigated the exposed cortex and vasculature with warm Ringer’s solution. Moreover, when the resection was close to major blood vessels irrigation with nimodipine was performed to potentially reverse or avoid vasospasm. As soon as MEP amplitudes stabilized or recovered, tumor resection continued. Moreover, when MEP failed to recover, resection was stopped at this part of the resection cavity.

### Post-hoc analysis of IOM data

Because a 50 % decline in amplitude was intraoperatively considered significant, all further data concerning surgical steps are related to this rule. A latency increase without simultaneous deterioration of amplitude was never seen in this series, so latency was excluded as a warning criteria. As all IOM data were routinely recorded and stored after every surgery, we were able to review these data post-hoc to determine the predictive value of different threshold values of substantial amplitude decline with respect to the patient’s postoperative status. Based on previous data of our and other groups, an amplitude decline of >50 and >80 % of baseline were therefore evaluated [2, 5, 6, 14, 31]. With regard to intraoperative data, incomplete reduction of compound muscle action potential (CMAP) was valued as deterioration rather than loss.

### Statistical analysis

For testing the distribution of several attributes, a Chi-square or Fisher Exact test was performed. Differences between 2 groups were tested using the Mann–Whitney-Wilcoxon test for multiple comparisons on ranks for independent samples. Differences between groups were tested by the Kruskall-Wallis test for nonparametric one-way analysis of variance (ANOVA) followed by Dunn’s test as the post hoc test. All results are presented as mean ± standard deviation (SD). Median and range were also calculated (GraphPad Prism 5.0c, La Jolla, CA, USA); *p* < 0.05 was considered significant.

## Results

MEP monitoring was successful in 53 metastases (92.9 %) and 105 gliomas (90.5 %). Table 1 presents specific details of enrolled patients such as sex, age, type of primary cancer in metastases and number, location of tumor, preoperative status, MEP changes, and surgery-related deficits.

### Postoperative results

**Fig. 2:**
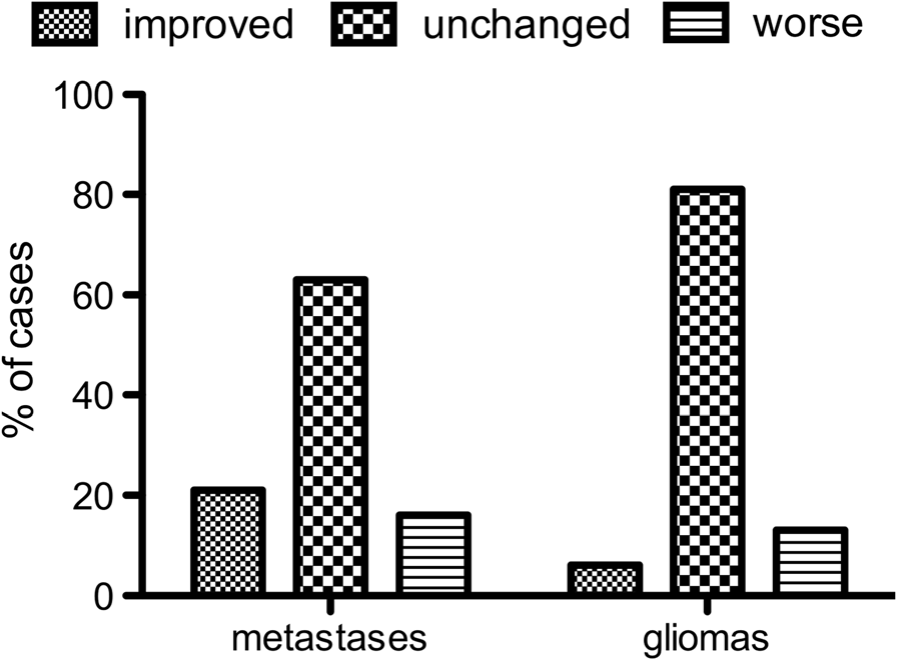
Differences in motor status. Differences in motor status during long-term follow-up between patients harboring metastases and gliomas (*p* < 0.05)

In general, both patients of each group suffered almost equally from a new permanent deficit due to surgery (BM 9.4 % vs. 12.5 % glioma), while 21 % of the BM group and 5.4 % of the glioma group showed improved strength due to surgery, 16.4 % of the BM group and 13.4 % of the glioma group worsened postoperatively (Fig. 2). Gross total resection (GTR) was intraoperatively estimated in the glioma group in 70.6 % (*n* = 77) and in the BM group in 92.5 % (*n* = 49) (*p* = 0.0013). Subtotal resection (STR) detected by postoperative MRI study was seen in 41.4 % of glioma patients (*n* = 46) and in 28.0 % of BM patients (*n* = 14) (*p* = 0.115). So we had to face an unexpected residual (UR) in 21.0 % of BM cases and in 12.0 % of glioma cases without showing statistically significant difference.

### Correlation of tumor type and location to postoperative deficit

WHO grade of the tumor did not play a significant role for postoperative temporary or permanent impairment of motor function (*p* = 0.6013), but a trend towards a correlation of high grade tumors and degree of postoperative deficit is shown. None of the patients harboring WHO grade I tumors developed a new surgery-related deficits. In the WHO grade II group all new deficits were only temporary in 17.6 % of cases (*n* = 3). Regarding high-grade glioma (WHO grade III and IV) temporary deficits occurred in 22.7 (*n* = 5) and 18.6 % (*n* = 13) of cases. Permanent deficits were seen in 13.6 (*n* = 3) and 14.3 % (*n* = 10) in these patients. Tumor location and postoperative temporary and permanent impairment of motor function showed statistically significant differences only in the BM group (BM: *p* = 0.0209 vs. gliomas: *p* = 0.6013).

### Intraoperative neuromonitoring

In 13 (3 BM and 10 gliomas) out of all 171 enrolled patients IOM was not possible. In a small number of cases displacement of the strip electrode occurred during resection with temporary stop of IOM or minor venous bleeding after removal of the strip electrode. There were no monitoring-related complications in all but one case, in which DCS caused an intraoperative focal seizure. Table 1 presents the intraoperatively judged stability of MEPs as graded by the neurophysiologist. The neurophysiologist gave constant information about MEP amplitudes and reported an amplitude reduction of more than 50 % of baseline to the surgeon. In all of these cases the surgeon temporarily stopped tumor resection, irrigated the surgical field, and/or released retractors. Results of IOM are given in the following and Table 2.

**Table 2:** False positive and negative results

		Gliomas			Metastases		
MEP decline		new deficits	temp	perm	new deficits	temp	perm
MEP decline >50 %	stable	4/9 (44.4 %)	3 (33.3 %)	1 (11.1 %/1B)	0/7 (0.0 %)	0	0
	reversible	19/85 (22.4 %)	14 (16.5 %)	5 (5.9 %/4B, 1I)	7/32 (21.8 %)	5 (17.2 %)	2 (6.9 %/1B,1 I)
	irreversible	10/11	2 (18.2 %)	8 (72.7 %)	3/14	0	3 (21.4 %)
	false positive	1	-	-	11	-	-
MEP decline >80 %	stable	5/24 (20.9 %)	4 (16.7 %)	1(4.1 %/1I)	4/30 (14.0 %)	2 (6.7 %)	2 (6.7 %/1B,1 I)
	reversible	18/70 (25.7 %)	13 (18.6 %)	5 (7.1 %/4B, 1I)	4/20 (20.0 %)	3 (15 %)	1 (5.0 %/1B)
	irreversible	10/11 (90.9 %)	2 (18.2 %)	8 (72.7 %)	3/3 (100 %)	-	2 (66.7 %/1U)
	false positive	1	-	-	1	-	-

B = Bleeding, I = Ischemia, E = Edema, U = Unknown; Showing all cases of false positive and negative results considering postoperative outcome in relation to postoperative MRI scan

### Post-hoc analysis: MEP reduction >50 %

The post-hoc analysis of MEP recordings revealed stable MEP-amplitudes in only 7 BM cases (13.0 %) with unchanged neurological status in all of these cases.

**Fig. 3:**
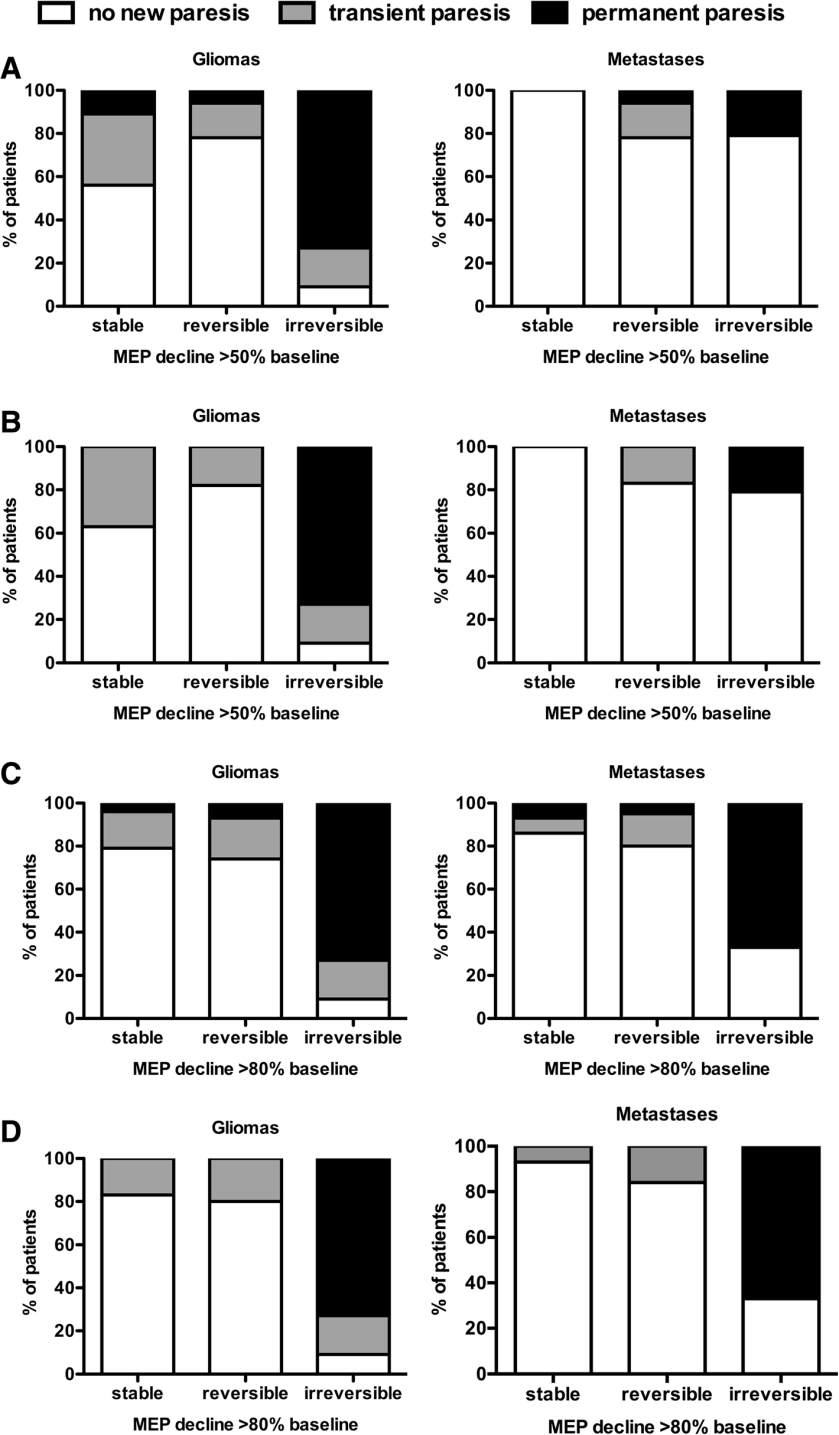
MEP reduction and surgery-related paresis. Intraoperative reduction of MEPs in relation to new postoperative impairment in motor function. Intraoperative MEP amplitude reduction is considered significant when exceeding 50 % (A & B) or 80 % (C & D). Parts A & C show the data with secondary events. Graphs B & D show cases without secondary events

We found stable MEP amplitudes in 9 glioma patients. In 5 cases (55.5 %) there was no change of postoperative status but in 4 other cases (44.5 %) we discovered new postoperative deficits, which not resolved in one case (11.1 %) (Fig. 3a).

#### Reversible decline >50 %

Out of 32 cases 25 BM patients (78.1 %) showed no new deficit. A new temporary motor deficit was found in 17.0 % (5 pts), and in 7.0 % (2 pts) motor function permanently deteriorated compared to the preoperative status. Both patients suffered from metastases within the precentral gyrus. In one case postoperative MRI revealed secondary postoperative hemorrhage and in the other patient an ischemic lesion was shown. Both presented with delayed paresis, which cannot always be detected by IOM. Thus, these events were considered to be secondary. Fig. 3c presents the data without those cases of secondary events. MEP amplitude reduction caused stop of resection in 6.0 % of these cases (2 patients). Postoperative MRI revealed residual tumor in one of these 2 cases.

In 85 glioma patients with reversible MEP decline 66 (77.6 %) remained their neurological status, while 19 patients developed transient (14 patients; 16.5 %) or permanent (5 patients; 5.9 %) new pareses. In these 5 patients suffering from permanent deficits MRI showed 4 secondary hemorrhages and one ischemia, which have to be seen as a secondary events and therefore not necessarily detectable by IOM (Fig. 3b).

#### Irreversible MEP decline >50 %

MEP recordings in BM cases showed an irreversible decline in 27.0 % (14 patients) of cases. No postoperative deterioration was observed in 11 patients, which represents false positive IOM. When investigating the time of continued IOM after amplitude decline, we found a range of IOM 2–27 min (median 4.7 min, mean 7.3 min) after >50 % decline. Maybe this period of IOM after MEP decline was too short to detect any MEP recovery. All but one patient harboring eloquently located gliomas and experiencing an intraoperative MEP decline of >50 % (*n* = 11) presented a new postoperative motor deficit. We found 2 temporary and 8 permanent pareses and had to deal with one false positive case (Table 2).

### Post-hoc analysis: Intraoperative MEP reduction >80 %

Regarding 80 % decline of initial MEP amplitude as considerable deterioration, we found stable results for IOM in 56.0 % (30 patients) of BM cases and in 22.9 % (24 patients) of glioma cases. In the BM group 86.0 % (26 patients) were neurologically unchanged in contrast to 7 % (2 patients) showing new temporary and 7 % (2 patients) new permanent deficit. One of the 2 patients with a new permanent paresis developed secondary hemorrhage postoperatively representing a case of false negative IOM at first glance. Out of the 24 glioma patients 19 (79.1 %) did not change postoperatively. Temporary deficit was seen in 4 cases and one patient developed permanent new deficit due to secondary ischemia in postoperative MRI, which might represent another case of false positive IOM. Figure 3c represents graph including secondary events.

#### Reversible MEP decline > 80 %

BM cases: In 20 cases (38.0 %) with reversible MEP reduction, 80.0 % (16 patients) kept their neurological status, 15.0 % (3 patients) showed new transient motor deficit, and 5 % (1 pt) worsened permanently. Glioma cases: in 70 (66.7 %) with reversible MEP reduction, the neurological status was unchanged in 74.3 % (52 patients), in 18.6 % (13 patients) temporary and in 7.1 % (5 patients) permanently worsened due to secondary hemorrhage in 4 cases and one case of ischemia.

#### Irreversible MEP decline >80 %

BM cases: We had 3 cases with irreversible MEP decline of which 2 cases developed a permanently new paresis and one case with no new surgery-related motor deficit. Again, this case has to be considered as a false positive case of IOM. We have to state that one reasonable explanation could be the time frame recording only 6.3 – 9.2 min (median 7.5 min, mean 7.5 min) after 80 % decline. Glioma cases: These 11 patients with irreversible MEP decline coincide exactly with the cases of 50 % MEP decline. Table 3 shows the receiver operating characteristics.

**Table 3:** Receiver Operating Characteristics

	Metastases		Gliomas	
Amplitude decline	>50 %	>80 %	>50 %	>80 %
true positive	6.0 %	4.0 %	8.0 %	8.0 %
true negative	70.0 %	89 %	84.0 %	84.0 %
false positive	21.0 %	2.0 %	1.0 %	1.0 %
false negative	4.0 %	6.0 %	6.0 %	6.0 %
negative predictive value	95.0 %	94.0 %	94.0 %	94.0 %
positive predictive value	21.0 %	67.0 %	73.0 %	73.0 %
sensitivity	60.0 %	40.0 %	57.0 %	57.0 %
specificity	77.0 %	98.0 %	97.0 %	97.0 %

Receiver Operating Characteristics (ROC) of MEP monitoring when an amplitude decline of >50 % or >80 % is considered significant depending on tumor type

### Intraoperative MEP loss

Out of 171 enrolled patients, 3 cases (2 BM and 1 glioma case) had complete intraoperative MEP loss. Both BM patients showed permanently new paresis. During surgery we had to stop resection in one patient of the glioma group due to MEP loss. Nevertheless the existing preoperative high-grade paresis improved significantly after surgery, so this case has to be considered as a false positive case of IOM. No further explanation was found even after thorough analyses of anesthesia protocols.

### Postoperative MRI scans

**Fig. 4:**
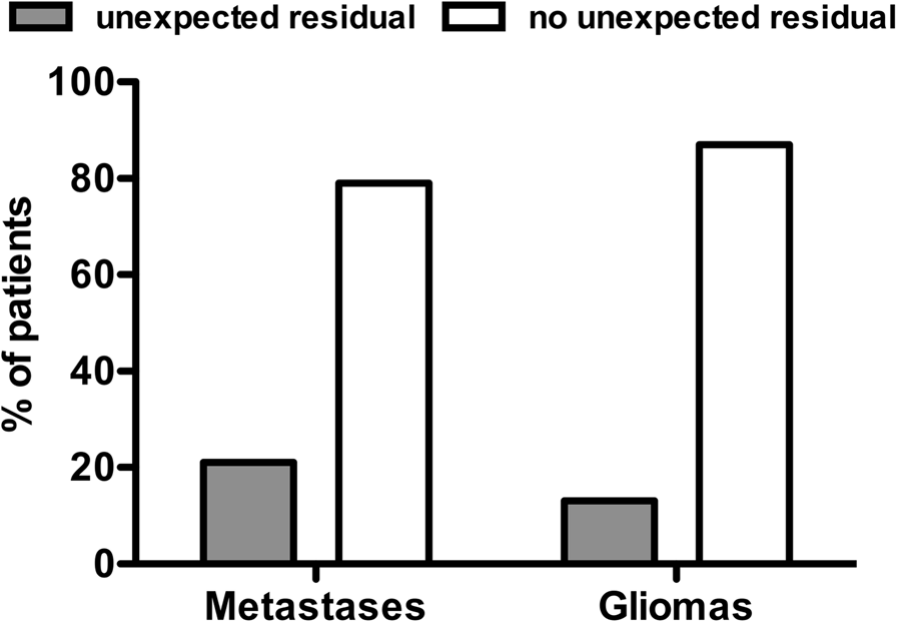
Unexpected residual. Postoperative MRI scan showing unexpected residual in both groups (*p* = 0.2265)

Considering postoperative MRI studies we were able to detect unexpected residual to the surgeon’s impression (Fig. 4) and to exclude secondary events for postoperative motor deterioration despite stable MEP amplitudes.

#### Tumor residual

UR was defined as the difference of the surgeon’s intraoperative impression of GTR and real GTR on postoperative MRI. In the BM group GTR was expected in 92.5 % and in the glioma-group in 70.6 %. MRI revealed STR in 28.0 % of all BM cases and in 41.4 % in of all glioma cases. Concerning suspected GTR, MRI revealead an UR in 21.0 % (BM) and 12.0 % (glioma) of cases (*p* = 144). Fig. 4 represents the unexpected residual (UR) in all enrolled cases (*p* = 0.226).

### Influence of IOM on the course of surgery

#### Extent of resection

In 54.7 % of all BM cases and in 65.2 % of all glioma cases, the neurophysiologist reported significant MEP deterioration of >50 % of baseline amplitude (Table 1). In cases of BM with stable MEP residual was found in 27.0 % on postoperative MRI, whereas STR was seen in 37.0 % in glioma cases (*p* < 0.05). Pausing of surgery due to MEP reduction or loss was caused in 34.8 % of all glioma-patients and in 8.0 % of BM patients (*p* = 0.0002). Resection had to be terminated in 15.2 % (17 patients) of glioma cases and 4.0 % (2 patients) of BM cases (*p* = 0.037). The incidence of new permanent motor deficit correlating with extent of resection was not statistically significant in both groups (STR: BM cases 25.0 %; glioma cases 11.4 % and GTR: BM cases 12.2 %; glioma cases 13.0 %).

#### False positive cases

In the BM group there were those 11 cases (22.0 %) in which irreversible MEP deterioration occurred during resection of the metastatic lesion without new surgery-related permanent deficit. In these cases postoperative MRI showed suspected residual tumor in 4 patients (36.4 %). In comparison, only 10 patients with STR out of the 50 remaining no false positive cases (20 %) were found (*p* = 0.0292). Regarding MEP decline >80 % we only had one false positive case without residual in postoperative MRI scan. Analysing the glioma data revealed different results. No matter whether 50 or 80 % MEP decline was used as cutoff, there was only one false positive case. This patient suffered from preoperative existing high-grade paresis due to left-sided postcentral GBM. Intraoperatively MEP loss caused stop of resection and motor deficit improved postoperatively. Postoperative MRI showed no residual tumor.

### Analysis of IOM characteristics

When analyzing MEP amplitude characteristics post-hoc, we also examined any amplitude reduction of more than 25 % as well as any latency prolongation of more than 10 %. Interestingly, there were differences between both groups. Regarding mean MEP reduction no statistical difference was found in both groups, whereas the mean time of reduced MEP only in the glioma group showed a significant difference between permanent and no new postoperative deficit (new permanent deficit: 445.1 ± 313.4 s [median 180.1 s; range 60.0 - 1800.0 s]; new temporary deficit: 100.1 ± 141.1 s [median 100.0 s; range 0.0 - 803.7 s]; no new deficit: 88.6 ± 64.3 s [median 80.6 s; range 0.0 - 421.3 s]; p = 0.001). Total MEP decline per recorded electrode did not reach statistical significance. But again, only in the glioma group mean time of MEP latency prolongation showed a significant difference between postoperative temporary or permanent motor deficit despite severe standard deviation (new permanent deficit: 142.1 ± 201.4 s [median 113.6 s; range 0.0 - 780.0 s]; new temporary deficit: 93.0 ± 186.3 s [median 40.0 s; range 0.0 - 816.3 s]; no new deficit: 43.2 ± 67 s [median 0.0 s; range 0.0 - 280.0 s]; *p* = 0.05).

## Discussion

In our study, both groups developed a new permanent motor deficit (BM 9.4 % vs. 12.5 % glioma), whereas the number of improved neurological status was much higher in the BM group (21.0 % vs. 5.4 %). These findings are in accordance to recent studies that reported similar results of motor function after resection of eloquently located lesions [2, 14, 17, 32]. We found a high number of UR with a trend towards a higher rate of UR in the BM group (21.0 % vs. 12.0 %; *p* = 0.144) without reaching statistical significance. Recently published studies could show a more infiltrative growth pattern of BM as expected in the past [1, 7]. The number of cases with estimated complete resection revealed a significant difference (*p* = 0.0013): In 93.0 % of BM cases GTR was expected by the surgeon in contrast to 72.0 % of GTR on postoperative MRI, which reflects the underestimation of real extent of BM.

### Correlation of tumor type, location, and postoperative pareses

In the glioma group we found no significant difference between WHO grade and surgery-related pareses, but a trend towards high grade tumors and degree of postoperative new paresis was shown, which can be related to a more aggressive resection of high-grade gliomas. In terms of tumor location only the BM group showed statistically significant differences (*p* = 0.0209), which seems not plausible at first glance. Regarding our patient cohort and its preoperative status (preoperative paresis in 57.0 % of BM vs. 31.3 % in glioma patients), pre-existing deficit is known to be a risk-factor for postoperatively aggravated paresis.

### Intraoperative neuromonitoring

The MEP evaluated by the neurophysiologist was more stable in glioma cases (65.2 % vs. 54.7 %). Based on different thresholds of MEP reduction we compared our results of BM cases with glioma cases in a post-hoc analysis. Contrary to the postoperative analysis of MEP recordings, the intraoperatively graded findings of the neurophysiologist show less stable MEP amplitudes during BM resection (Table 1). However, in the post-hoc analysis, MEPs were more stable in BM cases no matter what extent of MEP decline was taken. At a threshold of 80 % MEP decline we only had one false positive case instead of 11 cases at a MEP decline of 50 % (Table 2). As some other authors already stated, one reason could be the progressive depression of MEPs during general anesthesia [33].

As mentioned above, the median time frame of 7.5 min further recording after MEP decline could be a conceivable reason for missing a supposed MEP recovery. In the glioma group there was only one false positive case no matter what MEP decline was chosen to be considered. The other 10 of 11 cases with irreversible MEP decline all harbored high-grade gliomas and developed permanent (*n* = 8) and temporary (*n* = 2) pareses.

#### False negative cases

In both groups we found patients with suspected false negative IOM. All these cases could be explained by secondary events as revealed by postoperative MRI and no real false negative case was seen. Previous reports on false negative IOM are rare. Kombos et al. 2001 reported some false negative cases and Neuloh et al. described 2 patients with stable MEP recordings and new postoperative temporary mild facial palsy [32, 34]. Krieg et al. 2012 found no real false negative case in their study of predictive value of IOM in glioma surgery and all cases of motor deficits not detected by IOM could be explained by secondary events [2]. The result of no real false negative case of IOM is a very important information for the surgeon to believe in IOM.

#### False positive cases

Unfortunately we had to face 11 BM cases and one glioma case that had to be categorized as false positive cases when using MEP decline >50 % to be considered. Thus, the positive predictive value (PPV) of both groups differed considerably (Table 3). When using a MEP decline >80 % as threshold level, the PPV for BM approximated to the PPV of glioma cases, which shows that IOM is not the same in both groups and is more reliable in glioma cases, respectively. Comparing false positive cases with other BM cases we were most concerned about the significantly higher rate of STR in false positive BM cases. One explanation for this high rate of false positive IOM cases in BM patients could be that BM are moved within the brain parenchyma during resection and therefore causing some kind of mass effects in vasculature with transient subcortical hypoperfusion while gliomas are resected without moving the tumor and therefore causing less pressure on surrounding brain tissue. Another explanation may be the high rate of already existing preoperative pareses in BM patients. However, we have to keep in mind that only half of these 11 patients suffered from preoperative pareses. Worth mentioning that no matter which threshold was used for MEP decline, there was no difference of PPV for glioma patients. Yet, we could not find any reason for the one false positive glioma case in this retrospective setup.

### Influence of IOM on the course of surgery

**Fig. 5:**
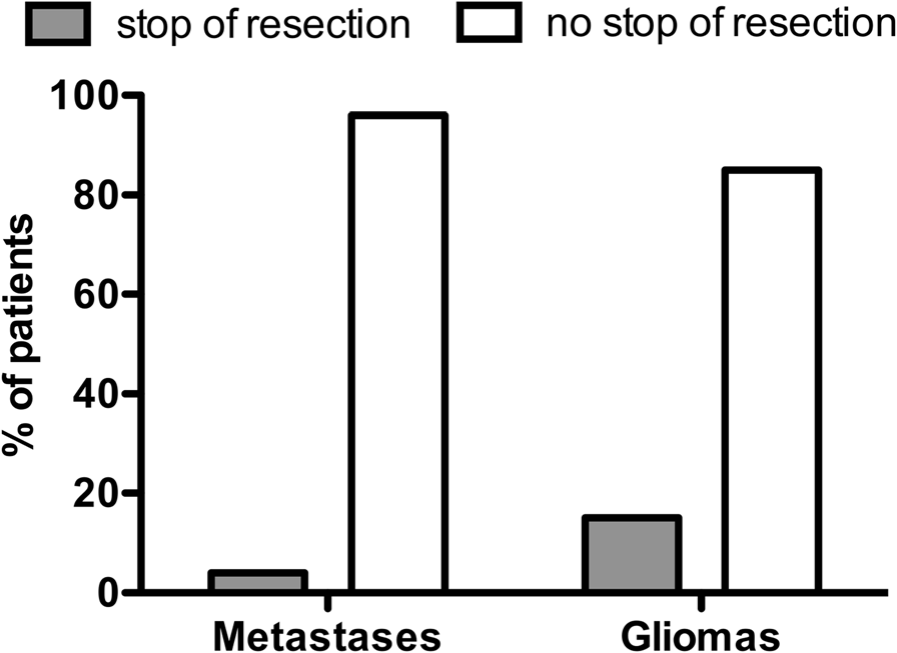
Stop of resection. Stop of resection due to decline in muscular evoked potential (MEP) amplitude below 50 % baseline in metastases and gliomas (*p* = 0.0375)

Pausing of resection due to MEP reduction or loss occurred significantly more frequently in glioma cases (*p* = 0.0002). The same result was found in cases where resection had to be stopped due to MEP decline (*p* = 0.0375) (Fig. 5). A trend of more STR in glioma cases was also seen which can be explained by the more infiltrative growth of pattern compared to BM. One can say that this indicates a negative influence of IOM on the extent of resection. However, Kombos et al. reported 2009 no negative impact of IOM on surgery of high-grade gliomas [14]. Other studies already showed a benefit of IOM during eloquently located lesions [32] and these findings seem to be reasonable since IOM may limit resection to prevent functional damage and to save quality of life which is the same intention of treatment in both groups.

### Analysis of IOM characteristics

We had to face a low rate of stable MEPs in both groups with a trend to more stable MEP recordings in gliomas as graded by the neurophysiologist. Yet, small changes of MEPs did not correlate with development of postoperative new paresis, which was stated by several studies previously [2, 16, 30, 34]. Furthermore a problem was the high rate of presumably false positive IOM cases of BM, if a threshold for MEP-decline >50 % was used. This indicates that different thresholds of MEP reduction have to be adapted to the patient’s lesion [14, 32, 34]. No matter what kind of lesion we were dealing with, IOM should be combined by diffusion tensor imaging fiber tracking, subcortical stimulation and navigated transcranial magnetic stimulation (nTMS). Furthermore monitoring of somatosensory evoked potentials can help us to estimate proximity of the CST, detect changes despite stable MEPs and increase the safety of resection [16, 27, 35, 36].

### Potential changes and postoperative outcome

IOM-related complications were very rare in this series, again pointing to the safety of IOM itself and the algorithms used in this considerably large series. All presumed false negative results in IOM can be explained by secondary hemorrhage or ischemia (Table 2). As some previous studies reported, if postoperative MRI scan showed no pathology, new postoperative motor deficit was always transient [2, 3]. We have to keep in mind that the shorter duration of follow-up in BM cases (0.7 months vs 6.1. months in glioma cases) maybe could not detect a recovery from permanent deficit. Apart from motor function, we still have to face the problem that we are unable to detect visual impairment or distinctive neuropsychological changes by only using cortical MEPs. Concerning the observed differences between IOM in metastases and gliomas the physiological or technical reasons are still not clear. Yet, this important issue has to undergo further investigation by a broad neurophysiological community.

### Limitations

In none of our cases intraoperative electrocorticography was used to detect intraoperative seizures as a confounding factor of the acquired IOM data. Senft et al. used multimodality such as intraoperative MRI combined with IOM to extend resection [37].

In BM cases stimulation of up to 20 mA and in one glioma case an intensity up to 30 mA was applied and therefore especially subcortical axons could be activated. In all these cases MEPs were stable and the patients developed no deterioration postoperatively. Routinely we used stimulation intensity of 6–12 mA.

We are aware of the fact that our median follow-up in BM group (0.7 months) does not allow defining a permanent deficit.

Most importantly, we also have to mention that the retrospective review of the patients’ outcome data might also have affected the results. Yet, due to the standardized neurological evaluation and follow-up of our patients, the loss of clinical information seems rather small. The short time of ongoing IOM after MEP decline or loss has to be considered, as maybe the rate of irreversible decline of MEP would have been lower.

Another major limitation of this study is that both groups differ in the number of lesions per patient, tumor location, follow-up, age, and baseline motor status (Table 1). However, due to the generally followed guidelines for indication of metastases resection, there is no option to provide two homogeneous groups to compare. Nonetheless, the differing baseline motor status might indeed be the reason for a significantly higher number of improved pareses in the BM group.

## Conclusion

By analyzing the results of MEP monitoring, we found a difference between glioma and metastases resection. MEPs in BM patients were more stable than in glioma patients in the post-hoc analysis. Moreover, the threshold for considerable MEP decline has to be chosen differently in glioma and BM surgery to reach the same level of reliability. Due to a surprisingly high rate of surgery-related new paresis in BM patients and therefore a remarkably higher rate of false negative IOM, an amplitude decline >80 % can be recommended for BM surgery, whereas this issue does no make any difference in glioma cases.

Right now, we do not know the reason for the observed differences. Thus, this report should motivate the IOM community to clarify this issue in order to provide improved care for our patients.
